# Marked effects of novel selective peroxisome proliferator-activated receptor α modulator, pemafibrate in severe hypertriglyceridemia: preliminary report

**DOI:** 10.1186/s12933-020-01172-8

**Published:** 2020-11-27

**Authors:** Chie Iitake, Daisaku Masuda, Masahiro Koseki, Shizuya Yamashita

**Affiliations:** 1 Iitake Clinic for Internal Medicine, 2131-1976 Migawacho, Mito City, Ibaraki 310-0913 Japan; 2 Department of Cardiology, Rinku General Medical Center, 2-23 Ourai-kita, Rinku, Izumisano, Osaka 598-0048 Japan; 3 Rinku Innovation Center for Wellness Care and Activities (RICWA), Rinku General Medical Center, 2-23 Ourai-kita, Rinku, Izumisano, Osaka 598-0048 Japan; 4 Department of Cardiovascular Medicine, Osaka University Graduate School of Medicine, 2-2 Yamadaoka, Suita, Osaka 565-0871 Japan

**Keywords:** Pparα, Spparmα, Pemafibrate, Severe hypertriglyceridemia, Hyperchylomicronemia, Familial chylomicronemia syndrome, Acute pancreatitis

## Abstract

**Background:**

Currently available treatments have only been partly successful in patients with severe hypertriglyceridemia, including those with high serum triglycerides above 1,000 mg/dL (11.3 mmol/L), who often suffer from acute pancreatitis. Pemafibrate is a novel selective peroxisome proliferator-activated receptor α modulator (SPPARMα) which has been developed as an affordable oral tablet in Japan. We herein report the first three patients with severe hypertriglyceridemia who were successfully treated with pemafibrate.

**Methods:**

Three patients with fasting serum triglyceride (TG) levels above 1,000 mg/dL (11.3 mmol/L) were treated with pemafibrate (0.2–0.4 mg/day, 0.1–0.2 mg BID).

**Results:**

Serum TGs decreased from 2,000–3,000 mg/dL (22.6–33.9 mmol/L) to < 250 mg/dL (2.8 mmol/L) without adverse effects in all three patients. Serum TGs in Patient 1 and 2 decreased from 1,326 mg/dL (15.0 mmol/L) to 164 mg/dL (1.9 mmol/L) and from 2,040 mg/dL (23.1 mmol/L) to 234 mg/dL (2.6 mmol/L), respectively. Patient 3 with type 2 diabetes and 12.1% (109 mmol/mol) hemoglobin A1c had a TG level of 2,300 mg/dL (26.0 mmol/L). Even after glycemic control improved, TG remained high. After pemafibrate administration, TG decreased to 200 mg/dL (2.3 mmol/L). All patients showed no serious adverse events.

**Conclusions:**

Pemafibrate demonstrated potential efficacy and safety for severe hypertriglyceridemia which may contribute to the prevention of acute pancreatitis, in a manner that can be easily prescribed and used as an oral tablet.

## Background

Treatment of patients with severe hypertriglyceridemia, in whom serum triglycerides (TGs) are > 1,000 mg/dL (11.3 mmol/L), is currently extremely challenging. The lowering of serum TGs to a reasonable level is crucial for preventing acute pancreatitis in patients with severe hypertriglyceridemia. Available treatments, including fibrates, niacin, and omega-3 polyunsaturated fatty acids, have only been partly successful. Clinical trials of fibrates have demonstrated some efficacy in patients with TG levels < 1,000 mg/dL (11.3 mmol/L) [1]. In a retrospective cohort study, most hypertriglyceridemic patients with a history of pancreatitis who were treated with fibrates had TG levels > 3,000 mg/dL (33.9 mmol/L) [2]. In these patients, rare dyslipidemic disorders are occasionally included. Familial chylomicronemia syndrome (FCS) is caused by mutations in the lipoprotein lipase (LPL) and related molecules. FCS is often underdiagnosed and unmanaged [3]. Currently available fibrates such as gemfibrozil, fenofibrate, and bezafibrate were shown to be ineffective for severe hypertriglyceridemic patients with LPL deficiency whose clearance of chylomicrons from the plasma is markedly impaired [4]. Treatment outcomes have been suboptimal in patients with severe hypertriglyceridemia, and data on treatment options are limited.

Pemafibrate [5, 6], a novel selective peroxisome proliferator-activated receptor α (PPARα) modulator (SPPARMα) developed by Kowa Co. (Tokyo, Japan), is currently marketed since 2018 only in Japan. It is available as an affordable oral tablet, which is in contrast with the latest gene-targeted therapies that are very expensive and generally administered via injections. The efficacy of pemafibrate in hypertriglyceridemic patients has been shown in clinical trials [6] and general practice [7]. In the phase 3 trial [6], pemafibrate at doses of 0.2 and 0.4 mg/day (0.1 mg and 0.2 mg BID) significantly reduced TG levels from the baseline by 46.2% and 45.9%, respectively, compared with a reduction of 39.7% achieved with the administration of 106.6 mg/day fenofibrate. Currently available fibrates are often associated with adverse events, such as worsening of renal function and elevation of liver enzymes levels [8, 9]. Fenofibrate was shown to increase alanine aminotransferase and gamma-glutamyltransferase levels, whereas pemafibrate significantly decreased both of them [6].

Treatment of severe hypertriglyceridemia for the prevention of acute pancreatitis is a significant challenge in patients with TG levels > 1,000 mg/dL (11.3 mmol/L). Episodes of relapsing acute pancreatitis can lead to chronic pancreatitis, as well as both exocrine and endocrine pancreatic insufficiency in the future [10]. But no study to date has verified the efficacy of pemafibrate in these patients. The PROMINENT trial, currently ongoing worldwide, aims to evaluate the efficacy of pemafibrate in preventing cardiovascular events in high-risk diabetic patients, although the trial excludes patients with TGs > 500 mg/dL (5.6 mmol/L) [11]. The present report of three patients with severe hypertriglyceridemia is the first ever evidence that pemafibrate can dramatically reduce very high serum TG levels > 1,000 mg/dL (11.3 mmol/L).

## Methods

### Patients

Patient 1 was treated in Rinku General Medical Center, Izumisano/Osaka, Japan. Patient 2, in Osaka University Hospital, Suita/Osaka, Japan. Patient 3, at Iitake Clinic for Internal Medicine, Mito/Ibaraki, Japan. All patients had a history of high serum TG levels > 2,000 mg/dL (22.6 mmol/L). Patients 1 and 2 were never medicated before, and Patient 3 partially responded to fibrate therapy in the past but suspended this treatment for a long time. Patient 3 also had a history of acute pancreatitis.

### Baseline treatment

All patients were placed on a fat restriction diet according to the Japan Atherosclerosis Society Guidelines for Prevention of Atherosclerotic Cardiovascular Disease 2017 [12] and had regularly scheduled visits with their doctors. Other fibrates, statins and omega-3 polyunsaturated fatty acids were not used before or during pemafibrate treatment.

### Pemafibrate treatment

The pemafibrate dose was determined by the treating physician. The standard pemafibrate dose was 0.2 mg/day (0.1 mg BID) and could be increased to 0.4 mg/day (0.2 mg BID). However, down-titration was considered for patients with liver dysfunction, as 0.1 mg once daily.

### Pharmacodynamic and genetic assessment

Laboratory data were collected from electronic patient health records. Blood samples were obtained under fasting conditions. Genetic testing was performed in Patient 2 and is currently under investigation in Patient 1. No genetic testing was performed in Patient 3.

## Results

**Fig. 1:**
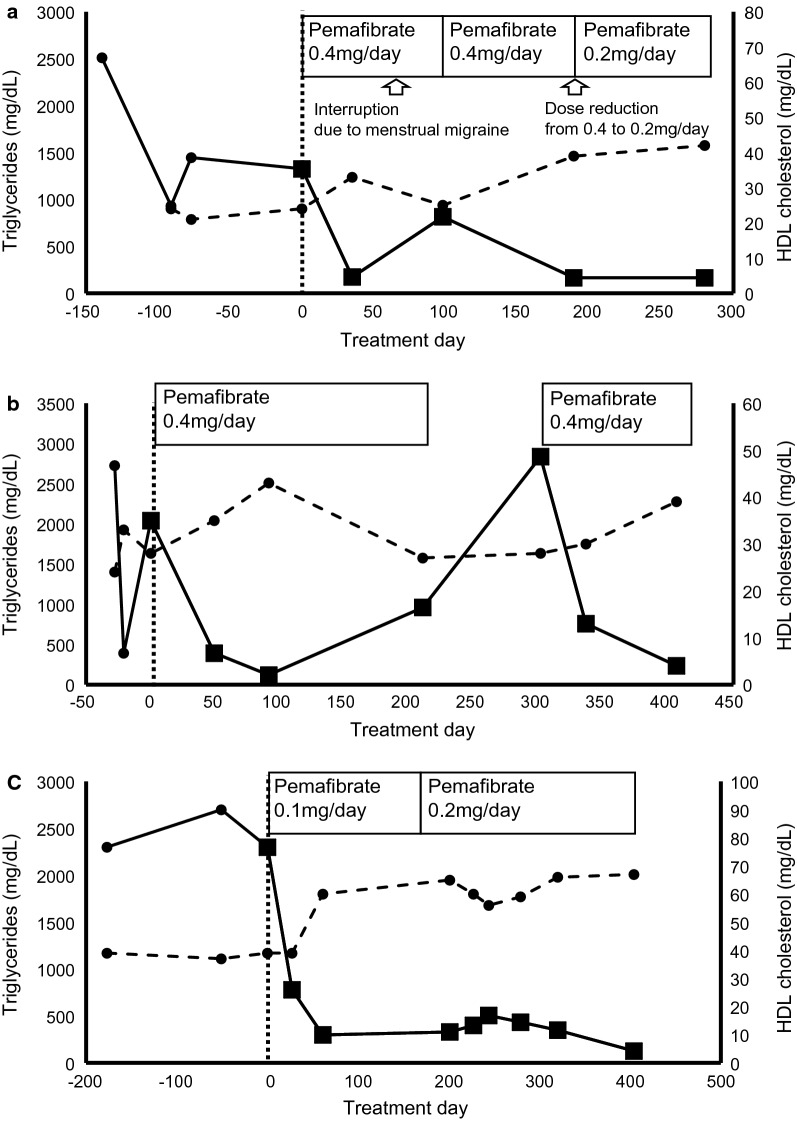
Effects of pemafibrate on serum triglyceride in patients with severe hypertriglyceridemia. **a** Patient 1, **b** Patient 2, **c** Patient 3. Day 0 is the baseline when pemafibrate was administered. In Patient 2, after the first administration, the patient was not compliant to take the drug. Nearly 200 days after the treatment, he suspended taking pemafibrate. Approximately after 300 days, he restarted to take pemafibrate regularly.  Triglyceride (mg/dL),  HDL-C (mg/dL)

Patient 1 (Fig. 1a) was a 49-year-old female who was diagnosed at a local clinic with severe hypertriglyceridemia at the age of 47 years. Her serum TG level was 2,511 mg/dL (28.4 mmol/L), and she had neither diabetes nor a history of acute pancreatitis. She was referred to Rinku General Medical Center. At the age of 30, her TG level was approximately 800 mg/dL (9.0 mmol/L) but was left untreated. She was an occasional alcohol drinker with a body mass index (BMI) 25.4 kg/m^2^. She was considered to have familial LPL deficiency based on a reduced LPL level. We are currently analyzing the genetic defect in Patient 1. Her mother also had severe hypertriglyceridemia and a history of acute pancreatitis, and her sister also had hypertriglyceridemia, suggesting that they may have a dominant genetic mutation.

Despite strict dietary fat restriction for 5 months, her serum TG levels exceeded 1,000 mg/dL (11.3 mmol/L). Thus, pemafibrate (0.4 mg/day, 0.2 mg BID) treatment was initiated. Her TG level decreased from 1,326 mg/dL (15.0 mmol/L) to 173 mg/dL (2.0 mmol/L) in one month. She discontinued pemafibrate due to a migraine attack during menstruation, which increased her serum TG level to 815 mg/dL (9.2 mmol/L). When she resumed pemafibrate, her serum TG decreased to 165 mg/dL (1.9 mmol/L). After 3 months, she reduced the pemafibrate dose to 0.2 mg/day (0.1 mg BID) because of misunderstanding, but her serum TG remained at 164 mg/dL (1.9 mmol/L).

Patient 2 (Fig. 1b) was a 21-year-old male diagnosed with hypertriglyceridemia at the age of 15 years in a nearby clinic. Due to worsening eating habits at the age of 19 years, his TG level increased to > 2,500 mg/dL (28.3 mmol/L) over 8 months. He was referred to Osaka University Hospital. He occasionally consumed alcohol and was not obese (BMI, 23.6 kg/m^2^). He did not have diabetes or a history of acute pancreatitis. We identified homozygous mutation of c.553G > T in *APOA5* gene, which was reported to be pathogenic and homozygotes manifest severe hypertriglyceridemia (mean TG = 2292 ± 447 mg/dL) [13]. Patients 2′s father was a heterozygote of this mutation and also had hypertriglyceridemia.

After 8 months of strict dietary fat restriction, pemafibrate (0.4 mg/day, 0.2 mg BID) was initiated. Serum TG level dropped from 2,040 mg/dL (23.1 mmol/L) to 392 mg/dL (4.4 mmol/L). However, he often missed taking pemafibrate, which led to elevated TG levels at 1,000–2,800 mg/dL (11.3 to 31.6 mmol/L). Nevertheless, his TG level was decreased to 234 mg/dL (2.6 mmol/L) after resuming regular pemafibrate treatment.

Patient 3 (Fig. 1c), a 43-year-old male diagnosed with hypertriglyceridemia at the age of 39 years in another clinic, was referred to Iitake Clinic for Internal Medicine with worsening diabetes. His fasting plasma glucose and hemoglobin A1c (HbA1c) levels were 297 mg/dL (16.5 mmol/L) and 10.3% (89 mmol/mol), respectively. He was diagnosed with type 2 diabetes. Dietary restriction and physical exercise were initiated. Despite initial improvement in laboratory parameters, he often interrupted the therapeutic lifestyle measures. When he returned 9 months after diagnosis, fasting plasma glucose, HbA1c, and TG levels were 239 mg/dL (13.3 mmol/L), 12.1% (109 mmol/mol), and 2,300 mg/dL (26.0 mmol/L), respectively. He had a history of acute pancreatitis, and liver dysfunction. He was consuming Japanese sake 2–3 alcohol units daily. His BMI was 28.2 kg/m^2^. His father had hypertriglyceridemia and type 2 diabetes.

Canagliflozin (100 mg/day) was initiated for glycemic control, and teneligliptin (20 mg/day) was added, which reduced HbA1c to 7.9% (63 mmol/mol) in 5 months. However, his TG level remained high at 2,300 mg/dL (26.0 mmol/L). A low pemafibrate dose (0.1 mg once daily) was initiated due to the history of liver dysfunction. After 1 month, his TG level dropped to 780 mg/dL (8.8 mmol/L). No adverse effects were observed, and pemafibrate was increased to 0.2 mg/day (0.1 mg BID). Serum TG level further decreased to 200 mg/dL (2.3 mmol/L), and HbA1c remained around 7.5% (58 mmol/mol). No correlation was noted between serum TG levels and glycemic control. For Patient 3, we could not perform a genetic testing.

Regarding the changes in serum HDL-C level, the improvement of serum TG level was generally associated with an increase in serum HDL-C level (Fig. 1) and a moderate increase in LDL-C level (Additional file 1: Figure S1). It is also very important to better characterize the safety of pemafibrate. Changes of liver and muscle enzymes, creatinine values as well as glucose and HbA1c are also shown in Additional file 1: Table S1. Pemafibrate was safe in terms of liver and kidney functions as well as glucose metabolism.

## Discussion

There are no effective therapies for severe hypertriglyceridemia at present. Alipogene tiparvovec (Glybera^Ⓡ^) [14], the first gene therapy for patients with familial LPL deficiency, was withdrawn from the market due to its extremely high cost and insufficient TG-lowering effect. Messenger RNA (mRNA) antisense and RNA interference approaches have been evaluated to modulate the levels of apolipoprotein C3 (apo C3), a potential new drug target for patients with FCS. Volanesorsen (ISIS 304,801, formerly INOS APOCIIIRx), a second-generation 2′-O-(2-methoxyethyl)-modified antisense inhibitor of apo C3 synthesis, was effective in three patients and reduced their TG levels to < 500 mg/dL (5.7 mmol/L) [15]. A recent study reported that antisense-mediated inhibition of hepatic apolipoprotein C3 (*APOC3*) mRNA with volanesorsen led to reduced plasma levels of apo C3 and TGs. Specifically, serum TG levels were reduced to < 750 mg/dL (8.5 mmol/L) in most patients with FCS [16]. Inhibition of angiopoietin-like protein 3 (Angptl 3) may be another target for FCS. Monoclonal antibodies (evinacumab, Regeneron Pharmaceuticals) [17], antisense oligonucleotides (AKCEA-ANGPTL3-LRx, Akcea Therapeutics; formerly IONIS-ANGPTL3-LRx) [18] and RNA interference (ARO-ANG3, Arrowhead Pharmaceuticals) [19] have been investigated. However, most of the new gene therapies are administered via injection and remain expensive, which hinder their applicability in many patients. Therefore, effective and cheaper strategies may be crucial for treating severe hypertriglyceridemia.

**Fig. 2:**
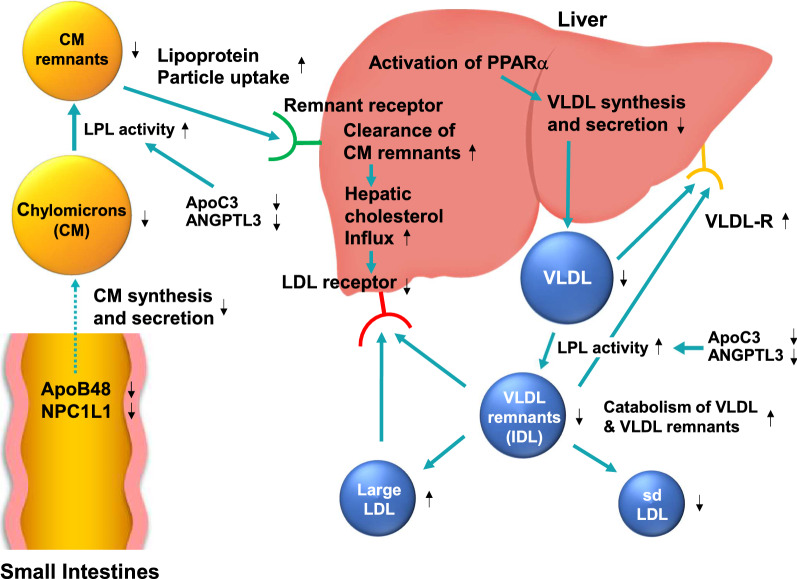
Possible mechanisms for pemafibrate on improvement of severe hypertriglyceridemia

Our three patients demonstrate the efficacy of pemafibrate, reducing TG levels from above 2,000 mg/dL (22.6 mmol/L) to below 250 mg/dL (2.8 mmol/L). Potential mechanisms underlying the effects observed in these patients are illustrated in Fig. 2. We speculate that underlying TG-lowering mechanisms may be distinct from those of conventional fibrates. Pemafibrate enhances LPL activity by PPARα-mediated increase in LPL synthesis and a reduction in apo C3, thereby promoting the catabolism of chylomicrons, very low-density lipoprotein (VLDL), and their TG-rich remnants. Our three patients did not have a complete, but only partial, deficit in LPL, which may be one of the reasons why pemafibrate was effective. However, we also speculate the possibility that there might be other pathways not mediated by enhanced LPL. In future studies, we could also test the effect of pemafibrate on serum TG levels in patients with complete LPL deficiency.

Pemafibrate enhances β-oxidation of fatty acids and suppresses the hepatic synthesis of TG and VLDL. Therefore, pemafibrate reduces not only serum TG but also chylomicron/VLDL remnants and small dense low-density lipoprotein (LDL) cholesterol, concomitantly increasing high density lipoprotein (HDL) cholesterol. Pemafibrate also inhibits the synthesis and secretion of chylomicrons from the small intestine [20]. It was also shown to suppress postprandial hyperlipidemia more strongly than fenofibrate in mice fed a high-fat diet by inhibiting mRNA expression of apolipoprotein B-48 (*APOB-48*), intestinal cholesterol transporter Nieman-Pick C1-like 1 (*NPC1L1*) and microsomal triglyceride transfer protein in the small intestine.

## Conclusions

The striking reduction in serum TG by pemafibrate may reduce the risk of acute pancreatitis in patients with markedly raised TG, which has not been achieved with currently available therapeutics. Moreover, pemafibrate is very affordable by prescription and is easy to administer as an oral tablet. Our experience from this proof-of-concept study in three patients may shed a new light into the management of patients with severe hypertriglyceridemia.

## Limitations

This is a small sample study of only three patients. Patients 1 and 2 had a transient suspension of taking pemafibrate, which may make the effectiveness of pemafibrate vague. However, after restarting pemafibrate, both cases showed a rapid reduction of TG, suggesting the sharp activation of PPARα by pemafibrate.

In our three cases, a genetic testing was performed only in one case (Patient 2) and homozygous mutation in the *APOA5* was identified. However, patients with extremely high serum TG levels may have a possibility of genetic disorders. Mutations of genes such as *APOC2*, *GPIHBP1*, *LMF1*, *APOA5* are already commonly known and it may be feasible to identify mutations in other two cases. However, inadequate lifestyle perhaps had a bigger influence on serum TG levels than genetic and polygenic mutations. In the future study, we may need to recruit more patients with hyperchylomicronemia and perform a case–control trial with genetic profiles.

## Supplementary information

**Additional file 1: Table S1.** Changes of serum lipids, liver function/renal function tests before and after treatment with pemafibrate.

## Data Availability

The datasets used and/or analyzed during the current study are available from the corresponding author on reasonable request.

## Abbreviations

APOA5: Apolipoprotein A5
APOB-48: Apolipoprotein B-48
APOC2: Apolipoprotein C2
APOC3: Apolipoprotein C3
FCS: Familial chylomicronemia syndrome
GPIHBP1: Glycosylphosphatidylinositol anchored high-density lipoprotein binding protein 1
HDL: High-density lipoprotein
LDL: Low-density lipoprotein
LMF1: Lipase maturation factor 1
LPL: Lipoprotein lipase
mRNA: Messenger RNA
Npc1L1: Nieman-Pick C1-like 1
PPARα: Peroxisome proliferator-activated receptor α
SPPARMα: Selective peroxisome proliferator-activated receptor α modulator
TG: Triglyceride
VLDL: Very low-density lipoprotein
