# Supplementary material for: Marked effects of novel selective peroxisome proliferator-activated receptor α modulator, pemafibrate in severe hypertriglyceridemia: preliminary report
Source: Cardiovasc Diabetol. 2020 Nov 27;19:201. doi: 10.1186/s12933-020-01172-8 (PMC7694943; doi:10.1186/s12933-020-01172-8)
Supplement: Supplementary file 1 — Additional file 1: Table S1. Changes of serum lipids, liver function/renal function tests before and after treatment with pemafibrate. [file 12933_2020_1172_MOESM1_ESM.pdf]

Additional Table S1. Changes of Serum Lipids, Liver Function/Renal Function Tests before and after Treatment with Pemafibrate.

A Patient 1

**treatment day**	**TG**	**HDL-C**	**LDL-C**	**blood glucose**	**HbA1c**	**AST**	**ALT**	**γGTP**	**ALP**	**CK**	**Cre**	**dose of pemafibrate**
day	mg/dL	mg/dL	mg/dL	mg/dL	%	IU/L	IU/L	IU/L	IU/L	IU/L	mg/dL	mg/day
-139	2511											
-91	932	24	41	95		16	11	19			0.6	
-77	1446	21	36			13	8	18				
0	1326	24	41			15	12	27				0.4
35	173	33	103									0.4
98	815	25	46	87		18	10	22		46	0.65	
189	165	39	121	94		17	12	18		51	0.62	0.4
280	164	42	125	88		21	16	21		54	0.6	0.4

B Patient 2

**treatment day**	**TG**	**HDL-C**	**LDL-C**	**blood glucose**	**HbA1c**	**AST**	**ALT**	**γGTP**	**ALP**	**CK**	**Cre**	**dose of pemafibrate**
day	mg/dL	mg/dL	mg/dL	mg/dL	%	IU/L	IU/L	IU/L	IU/L	IU/L	mg/dL	mg/day
-7	2725	24	62									
-21	392	33	146			21	14	34	209	247	0.82	0.4
0	2040	28	60									0.4
50	392	35	141			29	21	26	155	123	0.85	0.4
91	119	43	151	68		21	16	24	179	105	0.87	0.4
210	959	27	82	88		23	21	35	199	77	0.86	0.2
301	2837	28	70	108		29	27	54	224	117	0.77	
336	759	30	93			25	27	37	161	201	0.75	0.2
403	234	39	168			28	30	36	152	111	0.86	0.2

C Patient 3

treatment day	TG	HDL-C	LDL-C	blood glucose	HbA1c	AST	ALT	γGTP	ALP	CK	Cre	dose of pemafibrate
day	mg/dL	mg/dL	mg/dL	mg/dL	%	IU/L	IU/L	IU/L	IU/L	IU/L	mg/dL	mg/day
-177	2300	39	120	239	12.1	21	30	76	360	259	0.7	
-51	2700	37	100	180	9							
0	2300	39	110	178	8.1	47	56	146	316	247	0.78	0.1
27	780	39	149	123	7.9							0.1
61	298	60	248	128	7.3	17	15	43	184	112	0.54	0.1
201	330	65	152	118	7.9	22	24	80	202	165	0.76	0.2
227	400	60	134									0.2
244	505	56	139	153	7.7							0.2
279	434	59	121	159	7.9							0.2
320	349	66	175	124	7.9							0.2
404	127	67	127	98	7.7	17	13	35	193	138	0.87	0.2
